# Peroxiredoxin 6 phospholipid hydroperoxidase activity in the repair of peroxidized cell membranes

**DOI:** 10.1016/j.redox.2017.08.008

**Published:** 2017-08-12

**Authors:** Aron B. Fisher, Jose P. Vasquez-Medina, Chandra Dodia, Elena M. Sorokina, Jian-Qin Tao, Sheldon I. Feinstein

**Affiliations:** Institute for Environmental Medicine, Department of Physiology, Perelman School of Medicine of the University of Pennsylvania, Philadelphia, PA 19104, USA

**Keywords:** DPPP, diphenylpyrenyl phosphate, FA, fatty acid, FOX, ferrous xylenol orange, GPx, GSH peroxidase, PCOOH, 1-palmitoyl, 2-linoleoyl, *sn*-3-glycerophosphocholine hydroperoxide, LPCAT, lysophosphatidylcholine acyl transferase, PLOOH, phospholipid hydroperoxides, Prdx6, peroxiredoxin 6, PHGPx, phospholipid hydroperoxide GSH peroxidase, PMVEC, pulmonary microvascular endothelial cells, *t*-BOOH, *tert*-butyl hydroperoxide, WT, wild type, Lipid peroxidation, Oxidant stress, Hyperoxia, Endothelial cells, Perfused lung, Histidine mutation

## Abstract

Although lipid peroxidation associated with oxidative stress can result in cellular death, sub-lethal lipid peroxidation can gradually resolve with return to the pre-exposure state. We have shown that resolution of lipid peroxidation is greatly delayed in lungs or cells that are null for peroxiredoxin 6 (Prdx6) and that both the phospholipase A_2_ and the GSH peroxidase activities of Prdx6 are required for a maximal rate of recovery. Like other peroxiredoxins, Prdx6 can reduce H_2_O_2_ and short chain hydroperoxides, but in addition can directly reduce phospholipid hydroperoxides. This study evaluated the relative role of these two different peroxidase activities of Prdx6 in the repair of peroxidized cell membranes. The His26 residue in Prdx6 is an important component of the binding site for phospholipids. Thus, we evaluated the lungs from H26A-Prdx6 expressing mice and generated H26A-Prdx6 expressing pulmonary microvascular endothelial cells (PMVEC) by lentiviral infection of Prdx6 null cells to compare with wild type in the repair of lipid peroxidation. Isolated lungs and PMVEC were exposed to *tert*-butyl hydroperoxide and mice were exposed to hyperoxia (> 95% O_2_). Assays for lipid peroxidation in wild type control and mutant lungs and cells showed ~4-fold increase at end-exposure. Control lungs and cells showed gradual resolution during a post-exposure recovery period. However, there was no recovery from lipid peroxidation by H26A-Prdx6 lungs or PMVEC. These studies confirm an important role for Prdx6 in recovery from membrane lipid peroxidation and indicate that reduction of H_2_O_2_ or short chain hydroperoxides does not play a role in the recovery process.

## Highlights

• Repair of peroxidized lipids did not occur with H26A-Prdx6 Delete semicolons;mutation.
• Repair reflects the phospholipid hydroperoxidase and PLA_2_ activities of Prdx6;Move to next with "bullet mark" "P"eroxidase activity with small hydroperoxides and H_2_O_2_ does not play a role in repair.

## Graphical abstract

Conclusion: all 3 enzymatic activities of Prdx6 contribute to the reversal of cell membrane phospholipid peroxidation.

**Figure:**
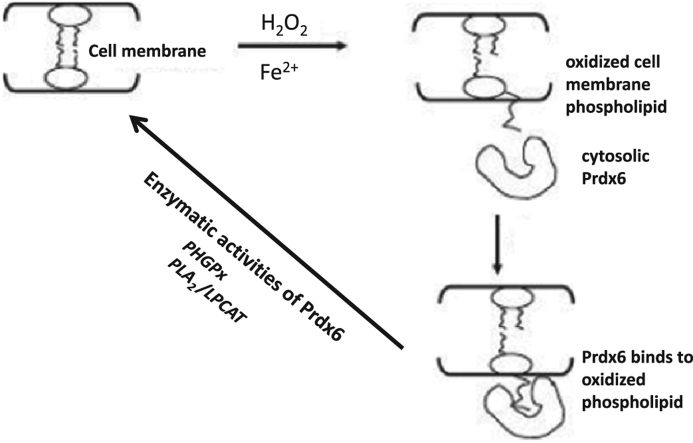

## 1 Introduction

Peroxidation of cell membrane phospholipids, one of the major manifestations of oxidant stress, represents a significant threat to membrane stability and commonly leads to cell death by apoptosis or a related mechanism [1], [2]. The accepted paradigm is that an oxidant stress initiates a chain reaction of lipid peroxidation that can be quenched by a tocopherol (*e.g.,*vitamin E) or some other chain-breaking anti-oxidant. However,those anti-oxidants do not reverse the oxidized phospholipids that already have been formed. While lipid peroxidation is not spontaneously reversible, enzymatic pathways for returning lipids to their reduced state have been described [1]. These pathways include reduction of the phospholipid hydroperoxide through peroxidase activity or hydrolysis of the phospholipid fatty acyl bond (PLA_2_ activity) followed by reacylation with a reduced fatty acyl CoA (lysophospholipid acyl transferase) [3]. Although these general pathways have been known for some time, the specific proteins involved in the repair of peroxidized phospholipids have been described more recently.

One of the reported repair proteins is glutathione peroxidase 4 (GPx4) that, unlike other members of the GPx family, directly reduces peroxidized phospholipids using GSH as the reductant [1], [4], [5], [6]. Although this enzyme has received considerable attention, reports of its tissue distribution indicate very low levels in many organs such as the lung [7], [8], [9]. Thus, this precludes an important physiological role in cell membrane repair, at least in those organs with very low levels of GPX4 expression.

Based on its physiological role, the lung is one of the vital organs most susceptible to oxidative stress. Our earlier studies showed that peroxiredoxin 6 (Prdx6) plays an important role in the prevention of lung lipid peroxidation and cellular injury associated with oxidant stress [10], [11], [12], [13], [14]. More recently, we have demonstrated directly that the anti-oxidant role of this enzyme can play a major role in the repair of cell membrane lipid peroxidation and cell survival [15], [16]. Prdx6 is widely expressed at significant levels in all tissues; it is a multifunctional enzyme that expresses both peroxidase activity and a coupled phospholipase A_2_ (PLA_2_)-lysophosphatidylcholine acyl transferase (LPCAT) activity [17], [18], [19], [20]. Similarly to GPx4, Prdx6 uses GSH as the physiological reductant [20], [21], [22], [23]. As indicated above, both the peroxidase and PLA_2_/LPCAT activities are considered as crucial in the repair of cell membrane lipid peroxidation [3]. Thus, Prdx6 could constitute a complete cell membrane repair protein.

Our previous studies of the direct role of Prdx6 in the repair of peroxidized cell membranes utilized mouse lungs, pulmonary microvascular endothelial cells (PMVEC) in primary culture, and cultured A549 epithelial cells.”Knock-out” of Prdx6 resulted in a decrease in the rate of peroxidized phospholipid repair [16], [24]. We further examined Prdx6 mutants that abolished one or the other activity of Prdx6. Mutation of Prdx6 that resulted in the inactivation of either its peroxidase (C47S-Prdx6) or PLA_2_ (D140A-Prdx6) activity resulted in a decreased rate of cell membrane repair following oxidative stress [16]; further,the absence of either activity resulted in increased cell death [15]. Thus, the PLA_2_ and the GSH peroxidase (GPx) activities of Prdx6 both play a significant role in the repair of peroxidized lung cell membranes.

The GPx activity of Prdx6 can reduce both short chain hydroperoxides, such as H_2_O_2_ or *tert*-butyl hydroperoxide (*t*-BOOH), as well as more complex phospholipid hydroperoxides (PLOOH) such as phosphatidylcholine hydroperoxide (PCOOH) [21], [25]. Clearly, the peroxidase activity of Prdx6 towards these two different substrates (H_2_O_2_ vs PCOOH) has very different implications regarding the role of the enzyme in oxidative stress. For example, reduction of H_2_O_2_ would prevent the formation of reactive O_2_ species (ROS) that are involved in biogenesis of lipid peroxidation while reduction of PCOOH represents a repair process. Our previous study evaluated the C47S mutant of Prdx6 [16]; this mutation results in the global loss of all GPx activity of Prdx6. The present study was designed to evaluate intact lungs and endothelial cells by comparing wild type (WT) with mutated Prdx6 (H26A); this mutation inactivates the ability of Prdx6 to hydrolyze oxidized phospholipids while preserving the peroxidase activity towards H_2_O_2_. Thus, the results of this study indicate the relative importance of the reduction of peroxidized membrane phospholipids vs reduction of short chain peroxide oxidants such as H_2_O_2_ in the cell membrane repair process.

## 2 Materials and methods

### 2.1 Materials

2-Thiobarbituric acid was purchased from Sigma-Aldrich (St. Louis, MO). *tert*-butyl hydroperoxide (*t*-BOOH) was purchased as a 70% solution from MP Biomedicals (Newport Beach, CA). Diphenylpyrenyl phosphate (DPPP) was purchased from Cayman (Ann Arbor, MI). Ferrous xylenol orange (FOX) was purchased as an assay kit from Northwest Life Science (Vancouver, Wa). 1-Palmitoyl, 2-linoleoyl, sn-3-glycerophosphocholine hydroperoxide (PCOOH) was prepared as a substrate for the peroxidase assay as described previously [21]. Recombinant wild-type Prdx6 protein was expressed in PET Blue-1 (Novagen, Madison, WI) and purified by column chromatography as described previously [24].

### 2.2 Mice

The use of mice for these studies was approved by the University of Pennsylvania Animal Care and Use Committee (IACUC). Male C57Bl/6 wild-type (WT) mice (8–10 weeks old) were obtained from the Jackson Laboratory (Bar Harbor, ME). Prdx6 null mice were bred in our animal care facilities. The generation and genotyping of these mice has been described previously [26]. The null mice have been fully back-crossed to the WT C57BL/6 background as confirmed using microsatellite marker analysis performed by the Jackson Laboratory (Bar Harbor,ME) [27]. The generation of H26A “knock-in” mice by the technique of recombineering has been described previously [19]; these mice were generated on the C57Bl/6 background. Through backcrossing, we have deleted the neomycin cassette and the presence of the flippase (FLP) gene from these mice.

### 2.3 Cells

Pulmonary microvascular endothelial cells (PMVEC) were isolated from the lungs of Prdx6-null mice as described previously [28]. The endothelial phenotype was routinely confirmed by fluorescence microscopy to determine cellular uptake of acetylated low-density lipoprotein labeled with 1,1′–dioctadecyl-3,3,3′,3′–tetramethylindocarbocyanine perchlorate (DiI-AcLDL) and by immunostaining with mouse monoclonal antibodies for PECAM-1 (Santa Cruz Biotech, Santa Cruz, CA) and vascular endothelial cadherin (VE-cadherin) (Abcam, Cambridge, MA) [28]. Prdx6 null PMVEC were infected by adding lentivirus prepared using primers as described previously to cells in growth media containing polybrene (∼8 μg/ml) [16], [19]. Cells were studied at 72–96 h post-transfection when expression of infected proteins was maximal. Empty HMD lentiviral transfer plasmid was used as a negative control.

### 2.4 Experimental procedures

Mice were anesthetized with intraperitoneal pentobarbital (50 mg/kg) and lungs were removed from the thorax for perfusion as described previously [16], [29]. The lung perfusate contained 3% fatty-acid-free bovine serum albumin and 10 mM glucose in Krebs Ringer bicarbonate buffer (pH 7.4); *t-*BOOH was added for the oxidant exposure. For oxidant exposure of PMVEC, *t*-BOOH was added to the incubation medium. For the hyperoxia experiments, mice were exposed to 100% oxygen in an exposure chamber (chamber O_2_ > 95%) as described previously [12], [16], [27]. CO_2_ absorbant and continuous airflow maintained the chamber [CO_2_] at < 0.3%.

### 2.5 Assays

Assay procedures are described briefly here; all procedures have been described previously in greater detail [16].

Lipid peroxidation was assayed by 3 different methods (TBARS, DPPP, and FOX). TBARS assay was used as a rapid screening procedure; it measures malondialdehyde (MDA) and other aldehydes that are predominantly generated from lipid hydroperoxides by the hydrolytic conditions of the *in vitro* assay, *i.e.,* high temperature and acidic medium [30]. Thus, the assay should reflect the *in vivo* reduction of lipid hydroperoxides. The DPPP and FOX reagents react directly with lipid hydroperoxides and therefore the change with time reflects the repair process [31], [32], [33]. DPPP was determined by fluorescence of the oxidation product (DPPP-ox).

TBARS were measured fluorometrically at an excitation wavelength of 530 nm and an emission wavelength of 550 nm. For the DPPP assay, cells or lung homogenate were incubated with DPPP (10 μM) in the dark at 4 °C for 30 min. Cell fluorescence was determined with the FLX800 microplate reader at 352 nm excitation and 380 nm emission wavelength; fluorescence of lung homogenates was measured at 380 nm (350 nm excitation) using a spectrofluorometer and values are reported as arbitrary fluorescence units (AFU). For the FOX assay, absorbance of the Fe^3+^- xylenol orange complex was measured at 560 nm with a spectrophotometer.

Prdx6-related enzymatic activities were measured for recombinant protein and for cell and lung homogenate. Total GSH-dependent peroxidase (GPx) activity was assayed by measuring the linear rate of NADPH oxidation in the presence of GSH plus GSH reductase [21]; this assay used H_2_O_2_ and t-BOOH as substrate. Phospholipid hydroperoxide GSH peroxidase (PHGPx) activity was assessed using the same assay with PCOOH as substrate. PLA_2_ activity was measured at pH 4 and 7 by the liberation of ^3^H-labeled free fatty acid from 1-palmitoyl, 2-[9, 10 ^3^H]-palmitoyl, *sn*-glycero-3 phosphocholine ([^3^H]-DPPC) [24], [29].

### 2.6 Statistical analysis

Data are expressed as mean ± SE. Statistical significance was assessed with SigmaStat software (Jandel Scientific, San Jose, CA). Group differences were evaluated by ANOVA followed by the Student *t*-test as appropriate. Differences between mean values were considered statistically significant at *P* < 0.05.

## 3 Results

### 3.1 Enzymatic activities

Enzymatic activities of Prdx6 were measured to confirm the effects of H26 mutation. Recombinant WT protein demonstrated roughly similar rates of GPx and PHGPx activities with *t-*BOOH and PCOOH substrates (Table 1). Mutation of His 26 to Ala had relatively little effect on the GPx activity of Prdx6 (~10% decrease, P > 0.05) but resulted in a marked decrease (92%, P < 0.05) of PHGPx activity. Results for activity with H_2_O_2_ substrate for recombinant protein (not shown) were similar to the results for *t-*BOOH. H26 is required for the binding of lipids to the protein, presumably the mechanism for the loss of PHGPx activity [34].

**Table 1:** Enzymatic activities of recombinant protein.

	aiPLA_2_ nmol/min/mg prot		Peroxidase μmol/min/mg prot	
	pH 4.0	pH 7.0	PCOOH	*t*BOOH
WT	104 ± 3	50 ± 2	4.94 ± 0.2	5.37 ± 0.3
H26A-Prdx6	1.0 ± 0.02*	0.3 ± 0.2*	0.38 ± 0.5*	4.92 ± 0.4

aiPLA_2_,acidic Ca^2+^-independent PLA_2_; PCOOH,phosphatidylcholine hydroperoxide; tBOOH, *tert*-butyl hydroperoxide; WT,wild type; values are mean ± SE for n = 3.

* p < 0.05 vs WT.

PLA_2_ activity also was present in recombinant Prdx6 (Table 1); activity measured at pH 4 was approximately double that at pH 7, as observed previously. PLA_2_ activity was essentially abolished by the H26A mutation. We ascribe the effect of H26 mutation on PLA_2_ activity both to lack of substrate binding as well as the loss of an essential component of the PLA_2_ catalytic triad [34].

Enzymatic activities also were measured in lung homogenate from WT, Prdx6 null, and H26A-Prdx6 mutant mice. The lung homogenate from WT mice showed significantly greater activity for GPx as compared to PHGPx (Table 2). This difference reflects the presence of other GSH-dependent peroxidases that can metabolize short chain hydroperoxides but cannot reduce PLOOH; an example is GPx type 1, the major GSH-dependent peroxidase in lung tissue. In lungs from Prdx6 null mice, the PHGPx activity was reduced by 96% while GPx activity was decreased by only 20% (Table 2), compatible with a role for other GSH peroxidases. The H26A mutation also abolished PLOOH GSH peroxidase activity (88% decrease, p < 0.05) while GPx activity was decreased by 10%. Results for activity with H_2_O_2_ substrate for recombinant protein (not shown) were similar to the results for *t*BOOH. PLA_2_ activity was essentially absent (98% decrease) in the lung homogenate of H26A–Prdx6 mice.

**Table 2:** Enzymatic activities of mouse lung homogenate.

	aiPLA_2_ nmol/h/mg prot	Peroxidase nmol/min/mg prot	
		PCOOH	*t-*BOOH
WT	8.79 ± 0.7	5.1 ± 0.1	48.3 + 2.1
Prdx6 null	0.01 ± 0.1*	0.2 ± 0.1*	38 7 ± 1.9
H26A-Prdx6	0.18 ± 0.1*^,^†	0.6 ± 0.3	43.5 ± 3.1

aiPLA_2_,acidic Ca^2+^-independent PLA_2_ measured at pH 4; PCOOH,phosphatidylcholine hydroperoxide; tBOOH,*tert*-butyl hydroperoxide; WT,wild type; values are mean ± SE for n = 3.

* p < 0.05 *vs* WT.

† p <.005 *vs* Prdx6 null.

Finally, enzymatic activities were measured in lentivirus infected PMVEC. The Prdx6 enzymatic activities for infection with vector alone reflect values for Prdx6 null PMVEC (Table 3). There is significant GPx activity but very low PHGPx and PLA_2_ activities in the null cells as for the whole lung homogenate from null mice. PMVEC from Prdx6 null mice infected with WT Prdx6 expressed the 3 activities of the protein that were measured—GPx,PHGPx,PLA_2_. However, infection with lentiviral vectors designed to express H26A-Prdx6 mutant protein resulted in cells expressing only GPx activity. Thus, the cells as well as lungs demonstrated the expected result of loss of PHGPx and PLA_2_ activities in the H26A-Prdx6 mutant.

**Table 3:** Enzymatic activities of lentivirus-infected Prdx6 null PMVEC.

	aiPLA_2_ nmol/h/mg	Peroxidase nmol/min/mg	
Vector (null)	0.07 ± 0.14*	1.0 ± 0.3*	135 ± 2*
WT	5.9 ± 0.2	12.4 ± 0.2	242 ± 1
H26A-Prdx6	0.01 ± 0.01*	1.2 ± 0.8*	217 ± 13

aiPLA_2_,acidic Ca^2+^-independent PLA_2_ measured at pH 4; PCOOH, phosphatidylcholine hydroperoxide; WT,wild type; values are mean ± SE for n = 3.

* p < 0.05 *vs* WT.

The LPCAT activity of Prdx6 (not shown) was measured in lungs and PMVEC by the previously described assay [19]; the H26A mutation of Prdx6 had no effect on this enzymatic activity and a possible effect of altered LPCAT activity on cell membrane repair was not studied.

### 3.2 Repair of lipid peroxidation

We evaluated the effect of the H26 mutation on recovery from lipid peroxidation in 3 models of oxidant stress.

#### 3.2.1 Isolated perfused lungs

**Fig. 1:**
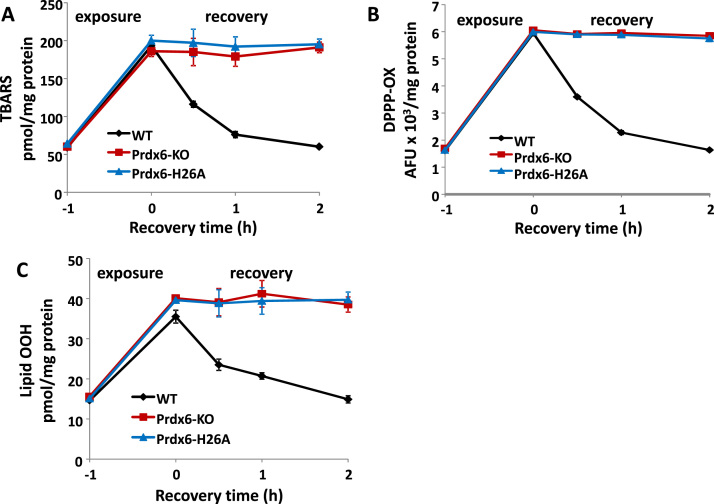
Repair of lung lipid peroxidation during perfusion of isolated mouse lungs.Lungs were perfused for 1 h (starting at −1) with medium containing 25 mM (WT) or 15 mM (Prdx6 null, H26A) *t*-BOOH followed by 2 h perfusion with fresh medium (recovery period). Experiments were terminated at intervals during the recovery period and lipid peroxidation in the lung homogenate was measured by assay of: A.TBARS; B. DPPP-oxide; and C. lipid OOH (FOX assay). Values are mean ± SE for n = 3 to 4.

In the first model, lungs that were isolated from wild type, Prdx6 null, and H26A “knock-in” mice were perfused with 25 mM *t*–BOOH (wild type) or 15 mM *t*–BOOH (Prdx6 null and H26A mutant). The differential concentrations of *t*–BOOH were selected so that the level of lipid hydroperoxides would be approximately similar at the end of the oxidant exposure for the WT and mutant lungs. After 1 h of oxidant exposure, the perfusate was replaced with *t-*BOOH–free medium and perfusion was continued for an additional 2 h (recovery). Lipid peroxidation was evaluated by measurement of TBARS, DPPP-ox, and the FOX assay at the end of oxidant exposure and at intervals during the recovery period. There was an approximately 3 to 4 fold increase in the level of lipid peroxidation after 1 h exposure to the oxidant for all 3 lung genotypes (WT,Prdx6 null,H26A-Prdx6) (Fig. 1). TBARS,DPPP-ox, and FOX assays all showed progressive return essentially to the control level of lipid peroxidation for the wild type lungs by the end of the 2 h recovery period. On the other hand, there was essentially no change in the degree of lipid peroxidation during the two hour post-exposure recovery period for lungs from either the the Prdx6 null mice or the H26A-Prdx6 knock in mice (Fig. 1).

#### 3.2.2 PMVEC

**Fig. 2:**
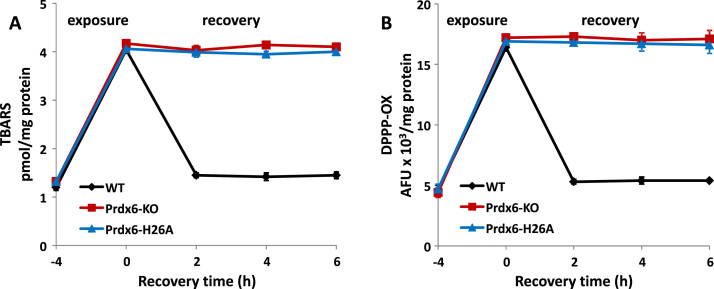
Repair of lipid peroxidation in pulmonary microvascular endothelial cells (PMVEC). PMVEC from Prdx6 null mice were infected with lentivirus to express vector alone (Prdx6 null), Prdx6 wild type (WT), or H26A-Prdx6.Cells were treated with 300 μM (WT) or 200 μM (null, H26A) t-BOOH for 4 h and then evaluated at intervals for recovery in medium free of *t*-BOOH by assay of: A. TBARS and B. DPPP-oxide.Values are mean ± SE for n = 3 to 4.

The second experimental model utilized Prdx6 null PMVEC that were infected by lentivirus to express wild type Prdx6, H26A-Prdx6, or vector alone (equivalent to Prdx6 null). These cells in primary culture were exposed to 300 μM *t*–BOOH (wild type) or 200 μM *t*–BOOH (H26A-Prdx6 and Prdx6 null) for 4 h. The medium was then changed to *t*–BOOH–free and cells were evaluated during a 6 h recovery period. Lipid peroxidation was evaluated by measurement of TBARS and DPPP fluorescence. Lipid peroxidation increased approximately 4-fold during the oxidant exposure period for all 3 cell types (Fig. 2). Cells expressing wild type Prdx6 showed return to control levels of lipid peroxidation by 2 h of recovery. No recovery was seen during 6 h post-oxidant exposure for the Prdx6 null or the H26A-Prdx6-expressing cells (Fig. 2).

#### 3.2.3 Exposure to hyperoxia

**Fig. 3:**
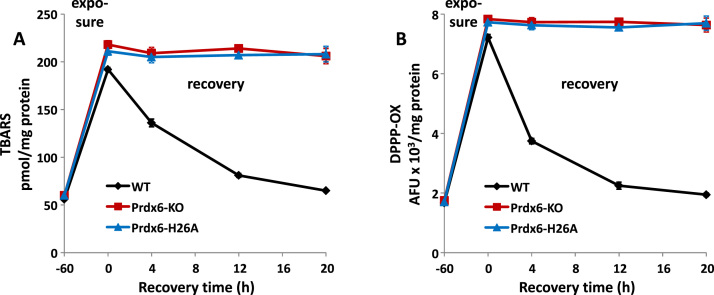
Repair of lung lipid peroxidation during recovery in room air following hyperoxia. Wild type (WT), Prdx6 null, and H26A-Prdx6 “knock-in” mice were exposed to > 95% oxygen for 60 h followed by a 20 h recovery period.Mice were sacrificed at intervals for analysis of lung lipid peroxidation by assay of: A. TBARS and B.DPPP-oxide. Values are mean ± SE for n = 3 to 4.

The third experimental model utilized mice that were exposed to > 95% O_2_ for 60 h; the ambient gas was then switched to room air and mice were evaluated during a 20 h recovery period. The duration of the O_2_ exposure period for mice was based on our previous experience for generating lung oxidative stress as an early manifestation of lung injury [12], [16], [27]. Lipid peroxidation was evaluated by measurement of TBARS and DPPP-ox fluorescence in the lung homogenate. Lungs from wild type, Prdx6 null, and H26A–Prdx6 knock-in mice were compared. The level of lipid peroxidation was increased approximately 4-fold at the end of the O_2_ exposure period in lungs from each of the 3 types of mice, although the increase of lipid peroxidation was slightly less in wild type compared to the other 2 conditions (Fig. 3). In wild type lungs, the level of lipid peroxidation gradually decreased during the recovery period and by 20 h essentially had returned to control levels. There was no change in the extent of lipid peroxidation during the recovery period for the Prdx6 null or the H26A–Prdx6 knock-in lungs (Fig. 3).

Thus,the presence of PHGPx and PLA_2_ activities of Prdx6 are essential for recovery from lipid peroxidation in mouse lungs and PMVEC while GPx activity *per se* (*i.e.,*reduction of H_2_O_2_ or short chain hydroperoxides such as *t-*BOOH) plays no significant role in the recovery process.

## 4 Discussion

This study evaluated the recovery of lung and PMVEC cell membranes from lipid peroxidation following exposure to an oxidizing stress. The relatively similar values for indices of lipid peroxidation in unexposed WT and mutant lungs and cells suggests that oxidative stress is minimal under the basal conditions of these experiments. The experimental oxidant stresses were *t-*BOOH administered to isolated lungs and cells *via* perfusion and exposure of mice to hyperoxia. These stresses resulted in cell membrane lipid peroxidation as indicated by measurements of TBARS and lipid hydroperoxides. TBARS measures breakdown products of lipid hydroperoxides that are formed largely *in vitro* during the course of the assay while the absorbance/fluorescence assays (FOX,DPPP) directly detect lipid hydroperoxides by oxidation of the fluorophore or by oxidation of Fe^2+^ to Fe^3+^ with binding to the dye xylenol orange [30], [31], [33]. At least theoretically, the lipid peroxides responsible for these reactions can be reduced back to their native state so that the measured extent of lipid peroxidation represents the balance between lipid oxidation and “repair”. The generation of isoprostanes from lipid hydroperoxides is a non-reversible reaction so this assay was not used in the present study since theoretically it would not reflect membrane repair.

Peroxidation of cellular lipids in the isolated lung was demonstrated during a 60 min exposure to *t-*BOOH. By adjusting the concentration of oxidant, the extent of lipid peroxidation at the end of the exposure period was similar for WT and mutant lungs. A similar strategy was used for study of cultured PMVEC. With wild type lungs and WT PMVEC, there was a gradual decrease in the levels of lipid peroxidation following removal of the oxidizing stress and values had returned to the control level within 2 h. The results for lung changes with the *in vivo* model of hyperoxia were essentially the same as for isolated lungs and PMVEC; there was significant lipid peroxidation at the end of 60 h of O_2_ exposure and a gradual return to control values during 20 h of post-exposure observation. On the other hand, there was no recovery from lipid peroxidation in lungs from Prdx6 null mice or Prdx6 null PMVEC during the period of observation, similar to our previously published results [16]. Similar to the results for Prdx6 null, there was no recovery from lipid peroxidation in the H26A-Prdx6 mutant lungs or cells. It is interesting that the time required for both oxidative injury and its repair in lungs from hyperoxia-exposed mice was significantly longer than seen with the isolated perfused lung; although this longer time for repair correlates with the longer time required for generation of the injury,as of yet, we do not understand the mechanism for the difference between the two models.

All peroxiredoxins including Prdx6 show peroxidase activity towards H_2_O_2_ and short-chain fatty acyl hydroperoxides [20], [35]. However, unlike the 2-Cys peroxiredoxins, Prdx6 also can reduce phospholipid hydroperoxides [21]. The present study was designed to answer the question as to whether the reduction of the oxidant,*e.g.,*H_2_O_2_ or *t-*BOOH, or the reduction of phospholipid hydroperoxides was responsible for the role of the Prdx6 peroxidase activity in recovery from lipid peroxidation. From a theoretical standpoint, reduction of H_2_O_2_ would not represent reversal of lipid peroxidation *per se* but would imply a residual oxidant stress that was neutralized by the peroxidase activity. This question was studied by the use of mice and cells expressing the H26A mutant as the only isoform of Prdx6. The H26 moiety is required for phospholipid binding to the protein and its absence results in loss of the ability to reduce phospholipid hydroperoxides [24], [34]. Additionally, H26A-Prdx6 loses PLA_2_ activity due to the role of H26 in the PLA_2_ catalytic triad as well as the lack of phospholipid substrate binding [34]. Reflecting these effects of H26 mutation, there was essentially no recovery from lipid peroxidation in the H26A-Prdx6 lungs and cells. These results confirm that the recovery from phospholipid peroxidation is not influenced by the presence of the GPx activity of Prdx6 to reduce small hydroperoxides.

The present results do not exclude a role for short chain hydroperoxides in the overall response of lungs to lipid peroxidation since other enzymes with peroxidase activity, besides Prdx6, are present in the lungs and cells. These other enzymes are presumably active under basal conditions (shown in part by the GPx assays) and can account for the lack of significant lipid peroxidation of the control tissues. However, our results show that these other peroxidases (for example, GPx1 and Prdx1 and 2) do not play a significant role in the repair of lung cell membranes. Indeed,we have shown that oxidative lung injury is significantly greater in mice with absent Prdx6 as compared with absent GPx1 [27]. While the present results do not differentiate between the phospholipid hydroperoxidase and the PLA_2_ activities of Prdx6, we have previously demonstrated that both activities contribute to the reversal of lipid peroxidation and both are required for maximal rates of recovery [15], [16]. Thus, the lack of any change of the level of lipid peroxides during the recovery period in the H26A-Prdx6 lungs/cells confirms the important role of these 2 activities in the repair of lung lipid peroxidation.

**Fig. 4:**
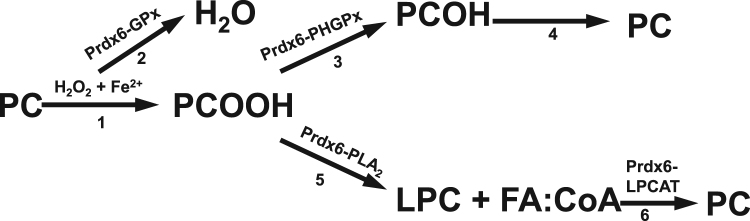
Schematic showing the role of Prdx6 in the pathways for oxidation and reduction of cell membrane phospholipids.The reactions are: 1) oxidation of the unsaturated fatty acid (FA) in phosphatidylcholine (PC) by Fe^2+^ catalyzed generation of ROS; 2) reduction (scavenging) of H_2_O_2_ by GSH-dependent GPx activity; 3) reduction by oxidized phospholipid by phospholipid hydroperoxide GPx (PHGPx) activity; 4) reduction of phospholipid alcohols by unspecified reductases; 5) hydrolysis of oxidized *sn*-2 FA (FAOOH) in PCOOH by PLA_2_ to generate lysoPC (LPC) plus an oxidized free FA (not shown); 6) reacylation of LPC with free FA:CoA by LPC acyl transferase (LPCAT) activity (the importance of the Prdx6-LPCAT activity in cell membrane repair has not yet been demonstrated experimentally). The net result is the regeneration of reduced phospholipid following an oxidative event.

The reactions for repair of phospholipid hydroperoxides associated with Prdx6 activities are shown schematically in Fig. 4. Phospholipid hydroperoxidase activity directly reduces phospholipid hydroperoxides to alcohols that can in turn be reduced by a variety of intracellular dehydrogenases; these latter reactions have not been investigated in any detail for lung phospholipids. In the second pathway for PLOOH repair, the PLA_2_ activity of Prdx6 removes the oxidized fatty acid to generate a lysophospholipid that in turn can by reacylated by the LPCAT activity of Prdx6; LPCAT enzymes other than Prdx6 could also play a role,although these are for the most part localized to endoplasmic reticulum, unlike the cytosolic Prdx6 [19]. The oxidized free fatty acid liberated from the oxidized phospholipid can be reduced by Prdx6, other peroxiredoxins/peroxidases, or metabolized by the mitochondria. Thus,all 3 activities of Prdx6--PHGPx,PLA2,LPCAT—have important roles in the reversal of membrane lipid peroxidation,and indicate that Prdx6 represents an essentially complete enzyme for the repair of peroxidized cell membranes.

## 5 Conclusions

Peroxiredoxin 6 plays an important role in the repair of peroxidized cell membranes. The key enzymatic activities of the protein are the ability to reduce and hydrolyze/reacylate peroxidized phospholipids. The ability to reduce H_2_O_2_ or short chain hydroperoxides does not play an important role in this process consistent with a lack of reports for effectiveness of the other members of the peroxiredoxin family in repair of lipid peroxidation.
